# Multiphysics Simulation for Efficient and Reliable Systems for Low-Temperature Plasma Treatment of Metals

**DOI:** 10.3390/ma19020382

**Published:** 2026-01-17

**Authors:** Nina Yankova Penkova, Boncho Edward Varhoshkov, Valery Todorov, Hristo Antchev, Kalin Krumov, Vesselin Iliev

**Affiliations:** 1 Faculty of Metallurgy and Material Science, University of Chemical Technology and Metallurgy, 1756 Sofia, Bulgaria; b.varhoshkov@ionitech.com (B.E.V.); todorov@uctm.edu (V.T.); hristo_antchev@uctm.edu (H.A.); kkrumov@uctm.edu (K.K.); veso@uctm.edu (V.I.); 2 Ionitech Co., Ltd., 1582 Sofia, Bulgaria

**Keywords:** plasma nitriding, glow electric discharge, vacuum chambers, electrohydrodynamics, conjugate heat transfer, numerical simulation, efficiency

## Abstract

Plasma nitriding is an advanced method to increase the hardness and wear resistance of different metal parts with complex shapes and geometries. The modelling is an appropriate approach for better understanding and improving such technologies based on multi-physical processes. Mathematical models of the coupled electromagnetic, fluid flow, and thermal processes in vacuum chambers for the low-temperature plasma treatment of metal parts have been developed. They were solved numerically via ANSYS/CFX software for a discretized solid and gas space of a plasma nitriding chamber. The specific electrical conductivity of the gas mixture, containing plasma, has been calibrated on the basis of an electrical model of the chamber and in situ measurements. The three-dimensional fields of pressure, temperature, velocity, turbulent characteristics, electric current density, and voltage in the chamber have been simulated and analysed. Methods for further development and application of the models and for technological and constructive enhancement of the plasma treatment technologies are discussed.

## 1. Introduction

The interest in plasma nitriding (ion nitriding) at glow electric discharge has been growing in recent years, as this technology allows for reliable, controllable and efficient processing of metal and alloy parts with different geometries [1,2,3,4,5,6,7,8,9,10,11,12,13,14,15,16]. The treatment is carried out in the electrically conductive gas space of vacuum chambers, where the partially ionised nitrogen interacts with the surface layers of the processed parts. Iron nitrides such as FeN, *ε*-Fe_2–3_N, and γ’Fe_4_N are formed as a result of complex chemical and physical processes on the metal surfaces, modifying their properties [2,3,14].

The increase in the surface hardness and wear resistance are important effects of plasma nitriding [2,3,4,5,6,7,8]. The surface hardness of stainless steels can be increased by 350–450% compared to non-nitrided steels [2].

The created nitride layers are oxidatively more stable than the base material. They increase the thermal resistance of the worn surfaces and prevent oxidative wear. The presence of a high-nitrogen phase with a hexagonal lattice reduces the coefficient of friction and the adhesion forces, improving the anti-seize properties of the surfaces [2,4].

Ion nitriding is also applied to increase the corrosion resistance of parts and tools [5,6,7,8]. In some cases, it can successfully replace thermal oxidation and some galvanic methods, such as zinc plating, cadmium plating, etc.

The above stated advantages are prerequisites for the increasing application of plasma nitriding for the processing of materials, used in mechanical engineering under extreme operating conditions, such as tools for metal processing in hot states (stamps and dies), tools for hammer dies, operating under shock loading conditions, presses, moulds for pressure casting, structural and stainless steels, etc. Improvements in the surface properties of the metal parts of electronic devices such as housings, fasteners, contact and mechanically loaded parts, moulds for manufacturing of plastic housings, and other elements for electronics with complex shapes are also possible with this technology.

**Figure 1:**
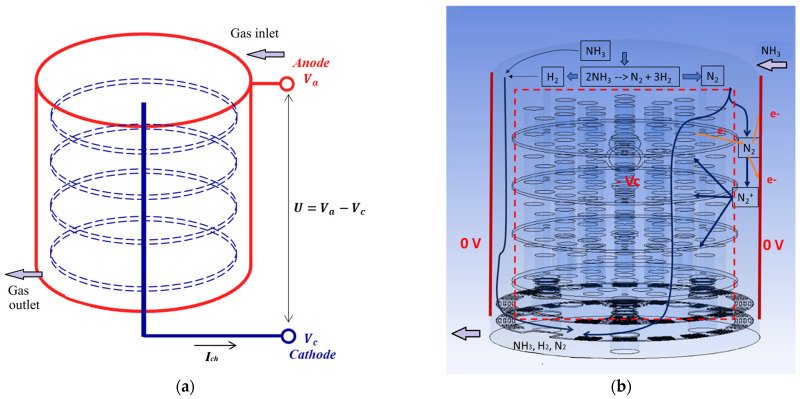
A vacuum chamber as a part of an electrical circuit (**a**) and a schematic view of the processes in the gas space under plasma nitriding (**b**).

The structure of the nitride layers depends on the maintained pressure, temperature, gas composition, and voltage in the chambers. The processed parts are connected as cathodes and the chamber walls are anodes in the electrical circuit (Figure 1a). A vacuum pressure inside the chamber and glow electric discharge, ignited by a pulse power supply, prevent high temperatures and the formation of an electric arc between the anode and cathode. The supplied gas is not pure nitrogen—it contains hydrogen in a certain ratio [2,3]. The studies show that the presence of H_2_ in the gases enhances the discharge current and increases the diffusion rate of nitrogen as a catalyst. Gaseous ammonia, which is chemically dissociated to N_2_ and H_2_ in the vacuum space, is often used as a cheaper variant [1,2,6]. The ammonia and the hydrogen can also partially dissociate into ions. However, the analyses of the positive ion content at plasma nitriding found that the nitrogen ions predominate in the gas space of the chambers [2,3].

The ionisation occurs at a closed electrical circuit, resulting in low-temperature electroconductive plasma (Figure 1b).

The N_2_ molecules are separated into positively charged ions and electrons as a result of the interaction with the electrons emitted by the cathode. The ions are accelerated towards the cathode and bombard the surfaces, increasing their temperature. This causes active emissions of electrons and knocking out of atoms from the cathode material. Part of these atoms (C, Fe, or other alloying elements) bind directly to the cathode surfaces with the active nitrogen, forming nitrogen-rich nitrides. Thus, the improved surface layers are a result of coupled electrical, chemical, mass transfer, and thermal interactions between the processed material and the gas mixture.

The knowledge of the multi-physical processes occurring in the vacuum space is important for the proper operation, improvement, and design of the plasma treatment system. Uniform concentration, thermal and electrical fields in the chambers at controlled temperatures, and electric current are necessary to achieve high-quality surface processing of all parts [2,10,14]. To maintain the desired temperatures, the chambers are equipped with additional heating and cooling systems that increase the cost of equipment, the electricity consumption, and the embodied energy of treated materials [2,15]. Energy savings and high-quality processing can be achieved by optimisation of

- Pressure in the chambers;
- Temperature of the processed parts;
- Supplied gas composition;
- Heat exchange through the chamber envelopes;
- Voltage in the electrical circuit;
- Duty cycle of the power supply;
- Time duration of the processing;
- Locations of the inlets and outlets of the gas mixtures;
- Rational utilisation of the workspace of the chambers.

Engineering knowledge and experience to improve and control the above processes and parameters in order to obtain predetermined properties of the surface layers have been established over time. However, the three-dimensional fields and gradients of the maintained variables in the vacuum chambers are uneven due to the complex shapes of the gas and solid media [16]. Higher gradients can lead to inaccuracy in automatic process control and uneven surface treatment of the parts in the workspace. Such problems can be predicted and fixed at the operation and design stages by modelling and numerical simulations of the transport phenomena in the ion nitriding chambers [17,18,19]. The published models cover the processes in the material, reflecting the thermal and mass interaction between the treated surfaces and the electroconductive gas via the boundary conditions. The influence of the nonuniformity of the electrical and thermal fields in the gas space on surface processing is not modelled. Coupled-field numerical simulations of the electromagnetic, chemical processes, and conjugate heat and mass transfer in the gas and solid domains of plasma nitriding chambers have not been published yet. The finite element analysis of electrohydrodynamics and magnetohydrodynamics (EHD and MHD) is an advanced approach to fill these gaps and to obtain detailed information about the fields and the gradients in plasma systems [20,21,22].

Mathematical models for the numerical simulation of the three-dimensional multiphysics phenomena during plasma nitriding, applicable for analysis and improvement in the efficiency of such technologies at the operation and design stages are reported in this paper. They build on the concepts and models presented in [13] regarding the radiation heat exchange between treated parts and the endothermic effect from the chemical reactions in the gas space. The models were tested and validated by numerical simulations of the processes in an industrial system for ion nitriding with high technological efficiency, determined by a refined control of the parameters in the chamber. This allowed for a more accurate analysis of the adequacy and precision of the models. Possibilities for their improvement and application for industrial systems are discussed based on the implemented numerical solutions.

## 2. Materials and Methods

### 2.1. Specifics of Industrial Plasma Nitriding

**Figure 2:**
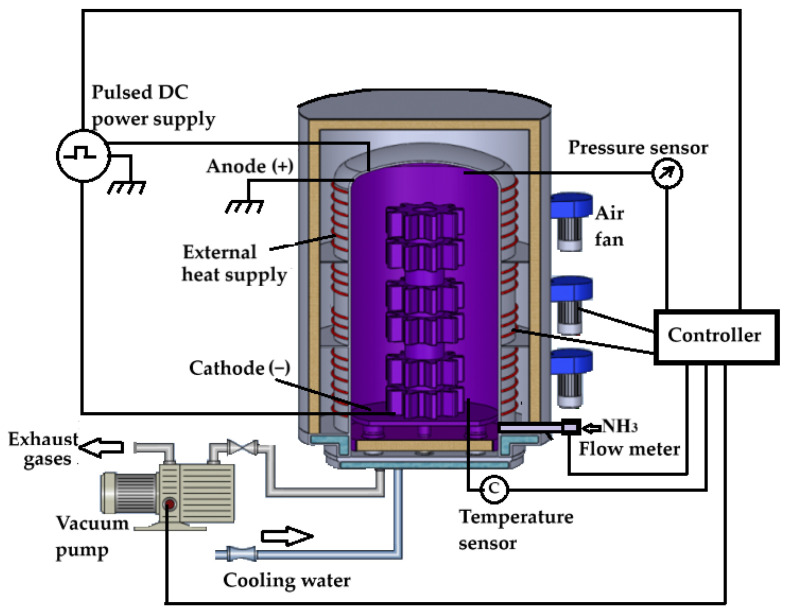
Plasma nitriding system [15].

**Figure 3:**
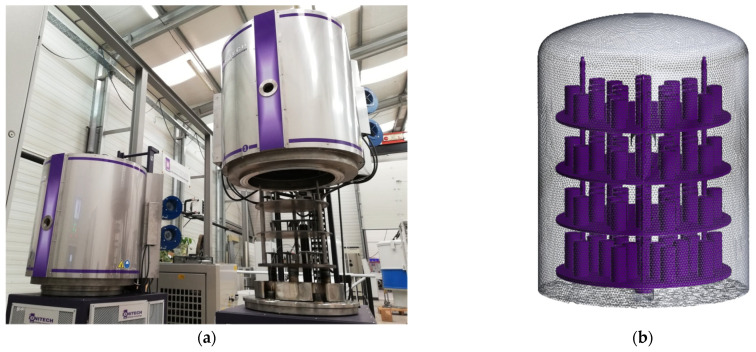
Hot-wall plasma nitriding system (**a**) and geometrical model of the chamber (**b**).

The advanced industrial systems for ion nitriding are equipped with a pulse power supply, a microprocessor to maintain the voltage and pulsation of the current, a gas flow meter, a vacuum pump to create a low absolute pressure, temperature sensors, and external heat supply systems to enable the set temperatures. A schematic and illustration of such a system, manufactured by Ionitech Co., Ltd. (Sofia, Bulgaria) [15], are shown in Figure 2 and Figure 3a.

The operation mode of the chamber is periodical. It is loaded with parts, arranged on metal shelves, and the power supply is turned on. Quasi-stationary fields in the chamber are reached after a few hours at controlled temperature, pressure, voltage, current, and duty cycles. This mode is maintained for a fixed time until the required surface properties of the processed products are achieved and the power supply is turned off. Details regarding the electrical equipment of the system and the electromagnetic processes are presented in [13]. It is established that such chambers can be considered as a predominantly active load with a low reactive component.

The absolute pressure in the chamber is maintained in the diapason of 100–500 Pa by a vacuum pump. The gas flow supplied to the chamber is monitored by a flow meter. The maintained temperatures on the cathode surfaces are usually between 480 °C and 600 °C, depending on the processed material. The vacuum chamber is thermally insulated and equipped with external heating and cooling sources. Their capacities are regulated comparing the measured and the set temperatures. This maintains precise temperature control of the treated surfaces. Deviation from the set temperatures by more than 14 K is not allowed, following the international standards for ion nitriding technologies. Detailed information about the construction and the maintained parameters is not possible due to confidentiality.

### 2.2. Mathematical Modelling and Numerical Simulation of the Multiphysics Phenomena in Vacuum Chambers for Ion Nitriding

In principle, the fields in the gas mixture containing plasma are electromagnetic. However, the magnetism can be neglected due to the lack of external magnetic forces in the modelled chamber and the processes in the fluid flow can be considered as electrohydrodynamic [13]. This hypothesis has been confirmed by initial numerical solutions of the models below, taking into account the magnetism and obtaining electromagnetic forces smaller than 1000 times or more than the buoyancy ones.

The generation of ions and their movement towards the cathode are not computed in detail, although their velocity is probably higher than the velocity of the non-ionised gas. These processes occur near the cathode surface and do not affect the gas flow caused by the vacuum pump. Furthermore, the ion concentration is relatively low, as it is found from the analysed chamber. The mass transfer on the cathode surface, resulting in iron nitrides, is also not included in the mathematical models, supposing that it does not significantly influence the conjugate heat transfer and the electrical processes in the chamber due to the relatively low amount of reactants and reaction products.

The presence of ions and electrons in the fluid space is modelled by defining it as a gas conductor. The mixture of ionised and non-ionised molecules is accepted as an ideal gas with non-zero electrical conductivity and a low magnetic permeability.

The electrical current between the anode and cathode through the electroconductive gas is computed by Ampere–Maxwell’s equations. The converted electrical energy into thermal energy during the energy interactions between the plasma and solid space of the processed parts is modelled by the Joule–Lenz effect [20].

Despite the low velocity of the non-ionised gases in the chamber, their flow is modelled as turbulent due to expected local turbulences during their motion through the solid parts arranged on shelves. This approach is increasing the accuracy of the solution for the velocity, pressure, and temperature fields by taking into account all essential and non-essential prerequisites for turbulence.

The heat exchange between the solid and fluid domain in the chamber is modelled as conjugate, computing the common temperatures and heat flows on the interface between the gases and solids by the solution of the energy equation. The heat transfer includes convection and radiation in the gas space and conduction in the solid shelves and processed parts. The gas mixture exchanges heat by radiation with the surrounding walls due to the non-zero absorptance and emissivity of the plasma and the molecules containing more than three atoms. These properties depend on the concentration of the ions, gas composition, and temperature [23]. Additionally, there is radiation heat transfer between the solid surfaces in the chamber “viewing” each other (surface-to-surface radiation). Such an energy exchange between the surfaces of the processed parts and the metal shelves is taken into account in the present study. The heat transfer through the chamber envelopes and jackets, containing heat sources, are modelled via the boundary conditions.

The endothermic effect in the case of the chemical dissociation of ammonia is modelled by a negative heat source in the energy equation, computed by the amount of dissociated gas per unit time.

In practice, the electrical fields in the chamber are quasi-stationary due to the pulse power supply. However, the time intervals of the pulsations are microseconds that impose short time steps and long computational times for transient analyses. In this study, all fields are accepted as steady state during the established mode of plasma processing to avoid long-term computations that may complicate the estimation of the model’s adequacy.

The governing system of equations is presented in Table 1. It is solved numerically by the finite volume method for the three-dimensional discretized spaces of the gas mixture, steel parts, and shelves via ANSYS 2024 R1 (ANSYS CFX) software. The boundary conditions are summarised in Table 2.

The initial conditions in the gas mixture and the solid domains are accepted near the expected variables in order to achieve a faster convergence at the iterative calculations. The initial voltage of the cathode is equal to the negative value of *V_c_* (Figure 1a). It was found that it does not change during the calculations.

The material models include

- The ideal gas relation for density calculations, specific heat, dynamic viscosity, thermal conductivity, the absorption coefficient, scattering coefficient, specific electrical conductivity, and magnetic permeability of the gas mixture in vacuum space;
- The density, specific heat, thermal conductivity, electrical conductivity, and magnetic permeability of the shelves and the processed parts.

The thermal properties of the fluid can be obtained at a known composition, pressure, and temperature of the electroconductive gas mixture [23]. The specific conductivity *σ* of the gas is the reciprocal of the specific resistance *ρ* [Ω·m] and can be obtained assuming the vacuum space as a hollow cylindrical conductor [13,24]:

(1) $$\sigma =\;\frac{1}{\rho }\;=\frac{l}{R\cdot S}\;S\cdot m^{-1},$$

where *R* is the electrical resistance of the gas conductor, determined by Ohm’s law at the known current *I* and voltage in the electrical circuit, Ω; *l* is the conductor length, with m and *S* being its cross-sectional area, m^2^.

The geometrical parameters *l* and *S* can be calculated approximately in complex gas spaces, which makes it difficult to determine *σ*. The latter can be calibrated iteratively with the numerical solution of the models above to achieve a computed electrical current, equal to the measured one in the operating circuit. The number of electrons per cubic meter reaching the anode per unit time and causing the electric current, can be obtained by

(2) $$C_{\alpha }=\frac{I}{e\cdot l\cdot S}\;\mathrm{electrons}\cdot m^{-3}\cdot s^{-1},$$

where *e* is the elementary charge of the electron: $e=1.602\;\times {\;10}^{-19}$ C (A·s).

This concentration is equal to the concentration of the positive ions at plasma equilibrium [25,26]. The number *α* of electrons and ions, reaching the electrodes per unit time, is

(3) $$\alpha =\frac{I}{e}\;\mathrm{electrons}\cdot s^{-1}\;(\mathrm{ions}\cdot s^{-1}).$$

The number of the gas molecules *N* in the chamber is

(4) $$N=1000\frac{m}{M}N_{A}\;\mathrm{molecules}\cdot s^{-1},$$

where M is the molar mass, kg·kmol^−1^; m is the mass of the gas, kg; and *N_a_* is the Avogadro number (*N_a_* = 6.02214076 × 10^23^ mol^−1^).

The number of gas molecules, supplied per unit time in the chamber, can be computed using the mass flow instead of the mass in Equation (4). The numerical information about the numbers of the ionised and non-ionised particles allow us to predict the share of the ions in the chamber and the heat flow due to endodermic reactions.

## 3. Results

The mathematical models above were solved numerically for the finite volume mesh, approximating the internal space of an operating chamber (Figure 3). The geometrical model accurately reflects the operating unit, but some elements of the solid bodies are confidential information and are not shown in Figure 3b. The boundary conditions were consistent with the measured and maintained parameters at operating mode in the chamber.

The material properties from ANSYS Library [20] are used to model the processed steel parts and shelves. The density of the gas mixture containing ammonia, nitrogen, hydrogen (products of the chemical dissociation), ions, and electrons is approximately equal to the density of ammonia at plasma equilibrium. Therefore, the fluid in the vacuum space is modelled as electroconductive ideal gas ammonia with vacuum magnetic permeability, an absorption coefficient of 1 m^−1^, a zero scattering coefficient, a refractive index of 1, and thermal conductivity of 0.1 W·m^−1^·K^−1^. The specific electrical conductivity was calibrated to obtain negligible differences between the measured current in the operating circuit and the computed one (3.5%). The latter is obtained as an integral of the nodal current density on the contact location on the cathode. A conductivity of 0.002 S·m^−1^ and equivalent conductor length *l* = 0.17 m were established during the calibration process. These are used to calculate the number of electrons and ions, reaching the electrodes per unit time (Table 3).

When the gas supplied to the chamber is NH_3_, the concentration of nitrogen molecular ions is higher compared to hydrogen, ammonia, and atomic nitrogen ions [2,16]. The minimum number of ammonia molecules that are chemically dissociated to generate nitrogen molecular ions is 2 × α, taking into account that one N_2_ and three H_2_ moles are obtained by the dissociation of two NH_3_ moles. If hydrogen and nitrogen atoms also ionise, the required amount of NH_3_ molecules that must dissociate to produce ions is less.

The calculations show that all ammonia molecules entering the chamber per unit time dissociate to generate ions. This is used to compute the heat flow during the endothermic dissociation in the energy equation.

If the ammonia concentration in the chamber is 100%, the number of gas molecules in it is determined by the NH_3_ mass according to (4). In the case of the chemical dissociation of all ammonia in the chamber, the number of the molecules is four times higher (last rows of Table 3). The share of the ionised particles per unit time is 1%.s^−1^ in the first case and lower in the second.

In practice, the concentrations of ammonia, nitrogen, hydrogen, ions, and electrons in the vacuum chamber are quasi-stationary due to the pulse power supply. The differences between the densities of N_2_, H_2_, and NH_3_ contribute to the buoyancy forces generated by gravity. Part of the hydrogen may be retained in the upper zone of the chamber because of the low velocities and lower density. The concentration of N_2_ in the bottom zone is expected to be higher due to its higher density. A similar distribution is established in [27]: the outlet gases during nitriding contain H_2_, N_2_, and NH_3_ with a higher N_2_ share when the supplied gas is NH_3_. The real gas concentrations and their fields in the vacuum space can be obtained by modelling a gas mixture of electrically conductive and electrically neutral particles.

The three-dimensional fields of the fluid flow, pressure, temperature, electrical, and heat transfer parameters, obtained as results of the numerical solution of the models, are presented in the figures below.

**Figure 4:**
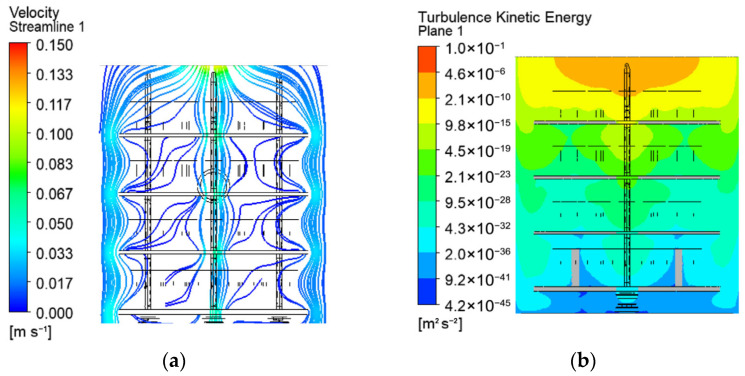
Gas streamlines around the processed parts (**a**) and turbulence kinetic energy in vertical cross-section of the workspace (**b**).

The streamlines of the gas mixture, visualising its flow, are shown of Figure 4a. Eddies and vortices are not established due to the low velocities in the chamber. The result is approximately zero turbulent kinetic energy of the gases (Figure 4b). Therefore, the gas flow in the chamber is nearly laminar. This is also indicated by the calculated low values of the Reynolds number (below 2320).

**Figure 5:**
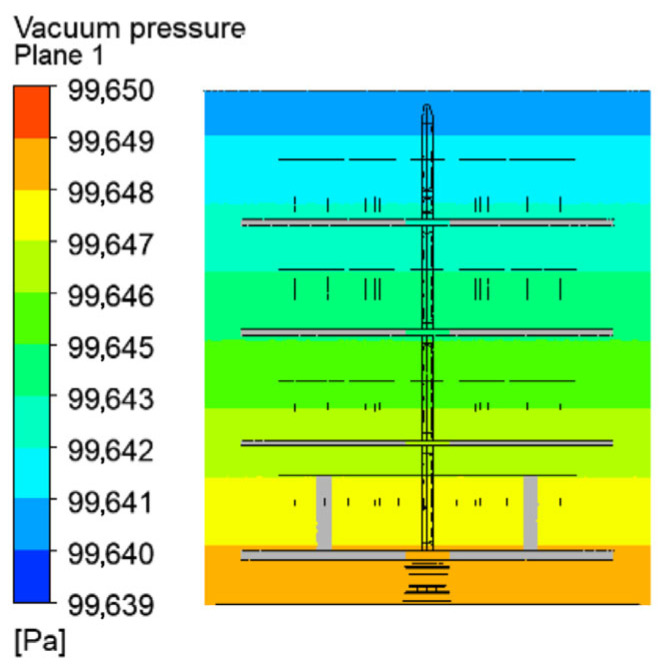
Vacuum pressure in a vertical cross-section.

The vacuum pressure, obtained as a difference between the atmospheric and absolute pressure, is shown in Figure 5. A vertical pressure difference of 11 Pa is established. The increasing vacuum pressure from the top to the bottom of the chamber is due to pressure drops in the flow direction.

**Figure 6:**
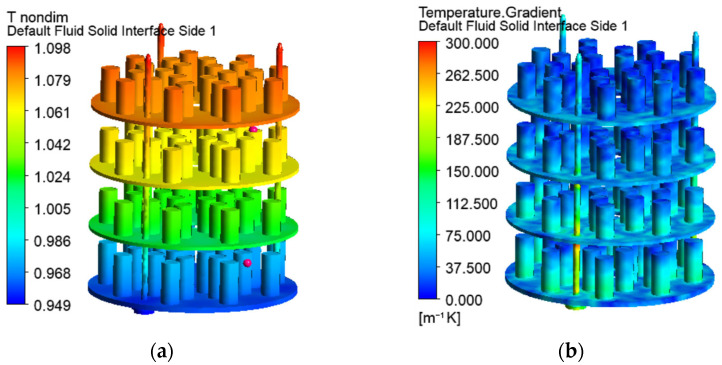
Nondimensional temperatures (**a**) and temperature gradients (**b**) on the surfaces of the metal parts and shelves.

**Figure 7:**
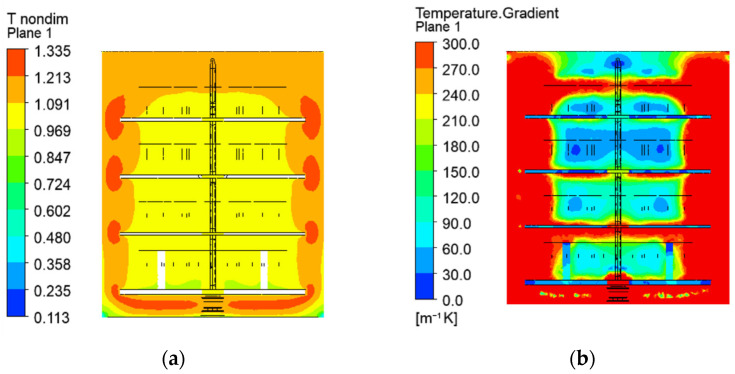
Nondimensional temperatures (**a**) and temperature gradients (**b**) in vertical cross-sections of the workspace.

Nondimensional temperatures, computed as ratios of the local temperatures *t* [°C] and the set temperature at the automation system *t_set_* (T* = *t*/*t_set_*), are shown in Figure 6a and Figure 7a.

Higher temperatures in the top rows of the processed parts (1.09 × *t_set_*) and lower in the bottom rows (0.96 × *t_set_*) are established. The nondimensional temperatures in the location of the temperature sensors, visualised by red dots in Figure 6a, are 0.974 (bottom sensor) and 1.064 (upper sensor). The maintained dimensionless temperatures in the operating chamber vary from 0.973 to 1.026. All computed values outside this range (including the dimensionless temperature at the location of the upper sensor) are not allowed in practice. Therefore, the simulated temperature stratification in the vertical direction exceeds the real one. The computed local temperatures of the processed parts in the second and third row are within the permittable limits. The temperatures of the parts on the upper and the bottom shelves are outside them. The maximum difference between the local computed and maintained temperatures is about 7%. That inaccuracy of the models is relatively high, taking into account the precise control of the temperatures in the operating ion nitriding chamber.

**Figure 8:**
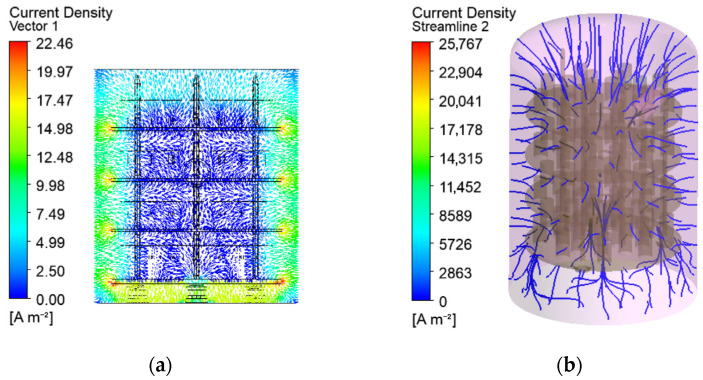
Current vector field in a vertical cross-section of the workspace (**a**) and current streamlines in the chamber (**b**).

**Figure 9:**
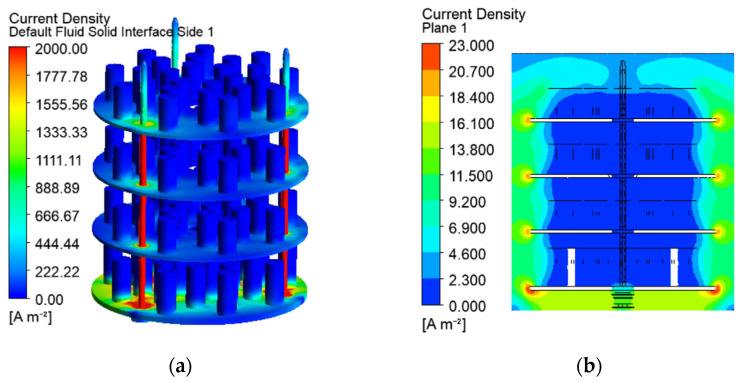
Current density on the solid surfaces (**a**) and in the gas domain (**b**).

**Figure 10:**
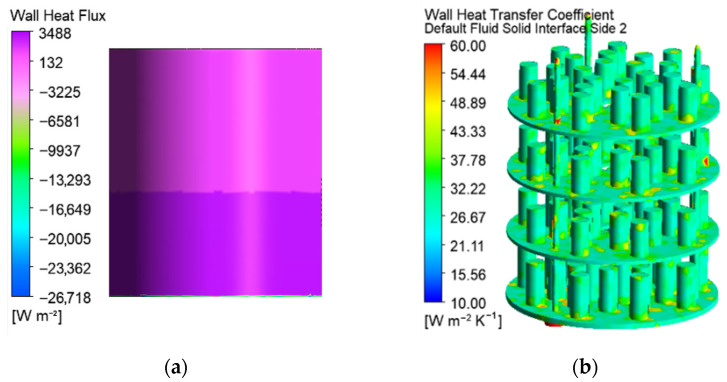
Wall heat flux on the internal walls of the workspace (**a**) and heat transfer coefficients on the solid surfaces (**b**).

The gas temperature between the shelves and the chamber walls in the vertical cross-section on Figure 7a reaches 1.34 × *t_set_*. These hot zones correspond to the locations of higher current density and electrical potential in the gas space, as shown in Figure 8 and Figure 9. The temperature near the walls depends on the heat transfer conditions, determined by the chamber envelopes. The exchanged heat flux varies with the vertical axis (Figure 10a). The integration of the nodal heat fluxes on the surfaces allows for obtaining of the input and output heat flows for an energy balance of the system.

Despite the changing heat exchange through the walls and the gas motion around the processed parts, the heat transfer coefficients between the gas and the treated surfaces of the metal parts are relatively uniform (Figure 10b). The radiation heat transfer in the gas space contributes to this positive effect.

The uniformity of the temperature field can be estimated by the temperature gradients, shown in Figure 6b and Figure 7b. They vary on the surfaces of the processed part and increase with the flow direction. The temperatures and their gradients are higher in the vertical supporting elements of the metal shelves, as they are pieces of the cathode and have a lower surface-to-volume ratio than the other metal bodies in the chamber. Although the heating of the support structure is not essential for the process, the higher temperatures and temperature gradients in them meet the expectations for the processing of bodies with complex geometries under industrial conditions [28].

Since the treated parts achieve uniform results in the analysed chamber, the computed temperature gradients are permittable.

**Figure 11:**
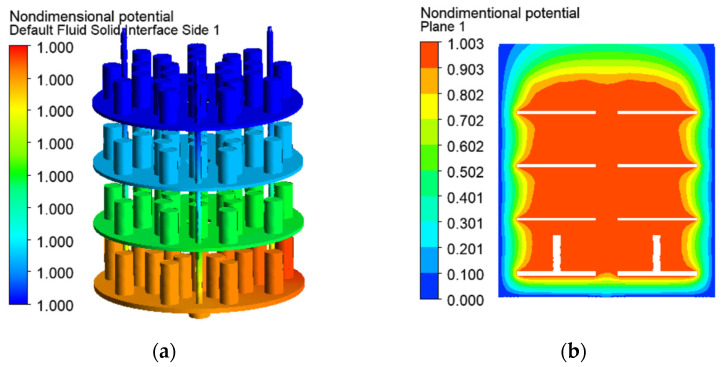
Nondimensional electrical potential on the solid surfaces (**a**) and in a vertical cross-section of the vacuum space (**b**).

The vectors and trajectories in Figure 8 show the probable direction of the electric current from the anode to the cathode surfaces as result of the electrostatic Coulomb forces. The electrical potential is visualised by nondimensional values, computed as ratios of the nodal potential *U* [V] and the maintained one *U_set_* in the operating system (U* = *U*/*U_set_*). The current density and the electrical potential are uniform in the processed parts due to their higher electrical conductivity and nonuniformity in the low-conductivity gas space (Figure 9 and Figure 11).

## 4. Discussion

The comparison of the local computed and maintained temperatures of the processed parts in the modelled chamber showed differences in the upper half of the workspace that reach 7% in some locations. This inaccuracy indicates weaknesses in the models of the radiation heat transfer, since the convective heat exchange has a smaller contribution to the temperature field due to the low velocities and laminar flow in the chamber. The models of the thermal boundary conditions are also subject to improvements by introducing codes for computing the heat sources as functions of the maintained temperatures, following the logic of the automation regulation in operating systems. For this purpose, it is necessary to simulate the non-stationary processes in the chambers while taking into account the pulse electrical supply.

More precise prediction of the transport phenomena in the vacuum space can be obtained by modelling the fluid in the chamber as a gas mixture of electrically charged and neutral particles. Additional models of the chemical dissociation of ammonia and the generation and motion of ions will allow for computing the concentration fields and properties, depending on the gas composition. Such an approach would be useful to specify the supplied ammonia flow and its distribution in the vacuum space in order to prevent it from entering the exhaust gases.

Positive aspects of the developed models have been identified despite the established inaccuracy and potential for improvements.

The numerical solutions in the present study show higher nonuniformity of the electrical and thermal fields in the gas space in comparison to the solid domain in the chamber (Figure 8, Figure 9 and Figure 11). The changes in the current density depending on the varying distance between the electrodes and the subsequent Joule–Lenze effect are possible reasons. Higher current densities in the zones between the nearest parts of the cathode and the anode and lower densities at the more distant electrode elements are established (Figure 9b). These differences are determined by the variable conductor length and subsequent resistances in the three-dimensional gas space. Therefore, the gradients of the electrical parameters in the gas conductor depend on its shape and vary with each change in the workload in the chamber, the geometry of the processed parts, and the way they are arranged on the auxiliary metal structure. The developed models are a suitable approach to predict the results of such changes and to prevent risks of arching at high-field locations.

Proper organisation of the fluid flow and heat transfer in the chamber to neutralise the above features of the electric fields are very important, taking into account that the surface temperatures and gradients determine the quality of processing. Variants at the design stage can be investigated by changing the geometry of the auxiliary metal structures for arranging the processed parts in the workspace. Uniform flow around the parts, radiation heat exchange between them, and rational utilisation of the workspace are criteria that are easy to estimate with the numerical solutions of the proposed models.

Local turbulizers of the auxiliary metal structures near the processed parts would reduce the temperature non-uniformity in the gas and improve the convective heat transfer. The presence of the turbulence model in the system of equations is important for the assessment of such effects.

Different methods of arrangement of the processed parts with complex geometry can be modelled and estimated based on computed electrical intensity, turbulence, convective, and radiative heat transfer in the vacuum space. Improvements in this direction can be achieved even in operating chambers with fixed geometry of the auxiliary metal structures.

The temperature uniformity of the processed surface can be analysed via the computing and visualisation of nondimensional nodal temperatures and temperature gradients in the workspace, as demonstrated in the previous section (Figure 6 and Figure 7). The nondimensional temperatures directly show the deviance of the local variable than the set one. Unallowable deviations can be predicted and avoided in the design and operating stages following the above guidelines.

Energy balances are easy to compute based on the heat fluxes through the chamber envelopes and the power consumption for plasma formation. The energy efficiency in different modelled scenarios is estimated by a comparison of the subsequent embodied energies.

## 5. Conclusions

The proposed approach for modelling the electrohydrodynamics and conjugate heat transfer during the plasma nitriding of metal parts enables a useful numerical study of different modes and configurations of the vacuum chambers during the design and operating stages. The prediction of the non-uniformity of the electric and temperature fields in the three-dimensional space of industrial plasma nitriding chambers at changeable workloads, processed parts, and operating parameters is the main advantage of such numerical simulations. Proper operation modes at desired set parameters without risks of poor quality of the treated parts and thermal problems in the units can be established hereby.

The application of the models for numerical simulation of the processes in an efficient industrial system allowed for a precise assessment of their accuracy and showed that they can be improved. Potential for future improvements has been identified in the following ways:

- Modelling of the chemical processes, generation, and movement of the ions in the gas space to obtain more detailed information about the coupled fields and gradients in the chamber;
- Modelling the transient transport phenomena in the chamber, taking into account the pulse power supply and automatic control of the heat transfer through the chamber walls.

## Figures and Tables

**Table 1:** Partial differential equations of the mathematical model.

Domain	Equations [20,21]
Electroconductive gas space	▪ Continuity equation ▪ Momentum equations, including electrical (Coulomb) forces ▪ Energy equation, including heat sources due to Joule–Lenz effect and endodermic chemical processes ▪ Shear stress transport (SST) turbulence model ▪ P1 radiation model of the radiative heat exchange between the electroconductive gas and the metal surfaces ▪ Electromagnetic model (Ampere–Maxwell’s equation)
Metal parts and shelves	▪ Energy equation for non-moving media, including heat source due to Joule–Lenz effect
	▪ Electromagnetic model (Ampere–Maxwell’s equation) ▪ Surface-to-surface radiation, based on Monte Carlo method for view factor computation

**Table 2:** Boundary conditions of the mathematical model.

Boundary	Boundary Conditions
Vertical walls (anode)	Zero voltage Zero velocities Robin boundary condition for the energy equation, including ambient temperature, heat transfer coefficient, and heat sources as functions of the coordinates in the vertical direction to model external heating and cooling Emissivity for radiation heat exchange with the gas mixture
Bottom (anode)	Zero voltage Zero velocities Dirichlet boundary condition: constant temperature of the cooling water Emissivity for radiation heat exchange with the gas mixture
Top (anode)	Zero voltage Zero velocities Robin boundary condition for the energy equation, including heat transfer coefficient, reflecting the thermal transmittance of the multilayer wall and the ambient temperature Emissivity for radiation heat exchange with the gas mixture
Gas inlet	Constant mass flux and temperature of the gas Zero electrical current density
Gas outlet	Constant absolute pressure, maintained by the vacuum pump Zero electrical current density
Insulated supports of the shelves	Zero electrical current density Emissivity for radiation heat exchange with the gas mixture
Contact location on the cathode	Constant voltage = −*V_c_* (Figure 1a)
Gas/cathode interfaces	Emissivity for radiation heat exchange with the gas mixture
	Zero velocities

**Table 3:** Numerical information about the molecules and ions in the investigated chamber.

Parameter	Value
Number of electrons and ions per cubic metre reaching the electrodes per unit time C_α_	1.95188 × 10^20^ particles·m^−3^s^−1^
Number of ions and electrons reaching the electrodes per unit time at plasma equilibrium	4.99376 × 10^19^ particles·s^−1^
Minimal number of dissociated ammonia molecules to produce nitrogen molecular ions	9.98752 × 10^19^ molecules·s^−1^
Share of dissociated supplied ammonia molecules	100%
Number of molecules at 100% ammonia in chamber (hypothesis)	7.1505 × 10^21^ molecules
Number of molecules after chemical dissociation of all ammonia molecules in chamber (hypothesis)	2.8602 × 10^22^ molecules

## Data Availability

The original contributions presented in this study are included in the article. Further inquiries can be directed to the corresponding author.
